# Sesbanimide R, a Novel Cytotoxic Polyketide Produced by Magnetotactic Bacteria

**DOI:** 10.1128/mBio.00591-21

**Published:** 2021-05-18

**Authors:** Ram Prasad Awal, Patrick A. Haack, Chantal D. Bader, Cornelius N. Riese, Dirk Schüler, Rolf Müller

**Affiliations:** a Department of Microbiology, University of Bayreuth, Bayreuth, Germany; b Helmholtz Institute for Pharmaceutical Research Saarland (HIPS), Helmholtz Centre for Infection Research, Saarland University Campus, 66123 Saarbrücken, Germany; c Department of Pharmacy, Saarland University, 66123 Saarbrücken, Germany; University of California, Berkeley; University of California, Berkeley

**Keywords:** glutarimide-containing polyketides, cytotoxic activity, *trans*-AT polyketide synthase, magnetotactic bacteria

## Abstract

Genomic information from various magnetotactic bacteria suggested that besides their common ability to form magnetosomes, they potentially also represent a source of bioactive natural products. By using targeted deletion and transcriptional activation, we connected a large biosynthetic gene cluster (BGC) of the *trans*-acyltransferase polyketide synthase (*trans*-AT PKS) type to the biosynthesis of a novel polyketide in the alphaproteobacterium Magnetospirillum gryphiswaldense. Structure elucidation by mass spectrometry and nuclear magnetic resonance spectroscopy (NMR) revealed that this secondary metabolite resembles sesbanimides, which were very recently reported from other taxa. However, sesbanimide R exhibits an additional arginine moiety the presence of which reconciles inconsistencies in the previously proposed sesbanimide biosynthesis pathway observed when comparing the chemical structure and the potential biochemistry encoded in the BGC. In contrast to the case with sesbanimides D, E, and F, we were able to assign the stereocenter of the arginine moiety experimentally and two of the remaining three stereocenters by predictive biosynthetic tools. Sesbanimide R displayed strong cytotoxic activity against several carcinoma cell lines.

## INTRODUCTION

Magnetotactic bacteria (MTB) share the ability to biomineralize membrane-enclosed organelles consisting of either magnetite (Fe_3_O_4_) or greigite (Fe_3_S_4_) crystals, called magnetosomes, which enable the cells to navigate within Earth’s magnetic field (1). Studies on MTB so far have focused mainly on understanding magnetosome structure, biosynthesis, and biological function as well as exploring the potential utility of magnetosomes as magnetic nanoparticles for various applications, such as magnetic imaging or magnetic hyperthermia, magnetosome‐based immunoassays, and as nanocarriers in magnetic drug targeting and multifunctional nanomaterials with versatile functional moieties (2–7).

Apart from their common ability to form magnetosomes, MTB represent a highly heterogeneous group of prokaryotes. They are abundant and widespread in the sediments of many diverse aquatic ecosystems, ranging from freshwater to hypersaline habitats (8, 9), and besides a multitude of free-living, single-celled MTB, multicellular and even ectosymbiotic members of this group have been discovered (10–12). MTB are known to have diverse and versatile lifestyles, and members of this group are found in many different classes of eubacteria (13–15). Within the last years, a wealth of genomic information has been obtained by conventional genomics, metagenomics, and single-cell genomics (14–20). We have recently shown that chances for the discovery of novel secondary metabolites clearly correlate with the increasing phylogenetic distance of the microorganisms under study (21). Because of their huge ecological, metabolic, phylogenetic, and genomic diversity, producers of such interesting natural products might also be expected among MTB. Indeed, Araujo et al. (22) first noted the presence of typical secondary metabolite biosynthetic gene clusters (BGCs), such as putative polyketide synthases (PKSs) and nonribosomal peptide synthetases (NRPSs), in the genomes of several MTB. However, this so far has remained an untapped source for discoveries, largely owing to the fact that most of these bacteria are not tractable; many cannot be cultured in the laboratory.

One of the few MTB that can be cultivated reasonably well and is genetically tractable is the alphaproteobacterium Magnetospirillum gryphiswaldense (23–26), which previously served as a model in many studies on magnetotaxis, organelle biosynthesis, and magnetite biomineralization (2, 27). Interestingly, several putative BGCs for secondary metabolites were tentatively predicted in its genome (22, 26). This prompted us to investigate in more detail the strains’ biosynthetic capability using a combination of molecular and analytical methods.

In this study, we focused on the role of a *trans*-acyltransferase PKS (*trans*-AT PKS) BGC in M. gryphiswaldense, which we identified as a homologue of the sesbanimide gene cluster described by Kačar et al. in parallel to our studies (28, 60). We set out to unambiguously assign the corresponding secondary metabolite from M. gryphiswaldense by markerless deletion of the gene cluster, to isolate the polyketide product and to elucidate its structure. Furthermore, we devised a model for sesbanimide biosynthesis that complements the one suggested by Kačar et al. (28, 60) and revealed the new sesbanimide R as a missing link between the sesbanimide biosynthesis pathways when compared across several taxa (29). In addition, we demonstrate cytotoxic activity of the novel sesbanimide congener.

## RESULTS AND DISCUSSION

### Identification, deletion, and transcriptional activation of a *trans*-AT PKS gene cluster.

Using the antiSMASH tool (30), we identified several secondary metabolite gene clusters in the M. gryphiswaldense genome. Three gene clusters were predicted to encode the biosynthesis of a putative lasso peptide, an aryl polyene, and a homoserine lactone (see Table S1 in the supplemental material). In addition, a large (69,942 bp) gene cluster was predicted to encode a putative *trans*-AT PKS. It has a conspicuously high G+C content (66.7% versus 63.2% of the entire genome) and comprises 30 open reading frames (ORFs), which were tentatively assigned to various constituents of a *trans*-AT PKS.

10.1128/mBio.00591-21.6 TABLE S1 Putative secondary metabolites gene clusters present in the genome of Magnetospirillum gryphiswaldense with locus tag and size. Download Table S1, DOCX file, 0.02 MB. Copyright © 2021 Awal et al. 2021 Awal et al. https://creativecommons.org/licenses/by/4.0/ This content is distributed under the terms of the Creative Commons Attribution 4.0 International license.

**FIG 1:**
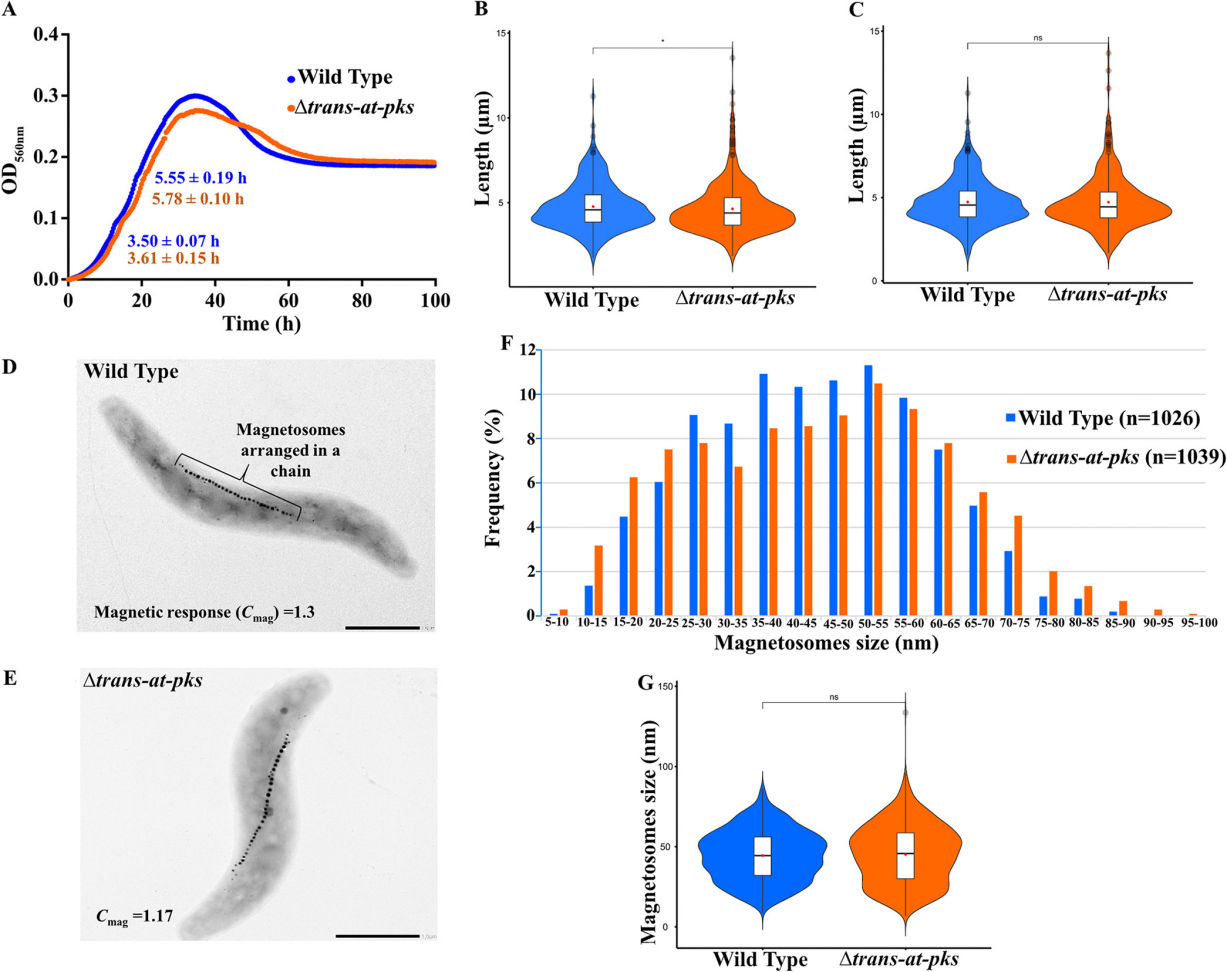
(A) Growth of the wild-type and Δ*trans-at-pks* strain under aerobic conditions where the target compound was produced. Each growth curve represents the average of two individual growth curves. The doubling time (*T_d_*) (mean ± SD) for each strain is given in the graph for the first and second part of the diauxic growth curve. (B) Cell length of the wild-type (mean = 4.77 ± 1.37 μm; *n* = 312) and Δ*trans-at-pks* (mean = 4.64 ± 1.46 μm; *n* = 504) strain grown under aerobic conditions. (C) Cell length of the wild-type (mean = 4.73 ± 1.37 μm; *n* = 347) and Δ*trans-at-pks* (mean = 4.72 ± 1.6 μm; *n* = 354) strain grown under microaerobic conditions. (D and E) TEM images of the wild-type (D) and Δ*trans-at-pks* (E) strain. (F and G) Analysis of magnetosome size distribution in the wild-type (mean = 44.45 ± 15.59 nm; *n* = 1,026) and Δ*trans-at-pks* (mean = 45.18 ± 18.29 nm; *n* = 1,039) strain.

To study the function of the cluster, we deleted the three putative core-biosynthetic genes (MSR-1_15630 to MSR-1_15650) encoding two large PKSs and a monooxygenase and a gene (MSR-1_15620) encoding an acyltransferase. The deletion comprised 41,295 bp and yielded a *Δtrans-at-pks* strain (Fig. S1). Growth of the Δ*trans-at-pks* strain was essentially wild type-like, with slightly increased doubling times during growth under aerobic conditions (Fig. 1A). Mutant cells were indistinguishable from the wild type with respect to length and shape (Fig. 1B to E). Cultures of the Δ*trans-at-pks* strain exhibited a lower magnetic response (*C*_mag_ = 1.17; wild type, *C*_mag_ = 1.3; *C*_mag_ is a light-scattering parameter for the semiquantitative estimation of average magnetic alignment of cells [31]). Transmission electron microscopy (TEM) of wild-type (Fig. 1D) and Δ*trans-at-pks* (Fig. 1E) cells showed that the strains formed magnetosomes in about same numbers and with similar average sizes (Fig. 1F and G); however, both smaller (<25 nm) and larger (>60 nm) particles were more frequent in the Δ*trans-at-pks* strain than in the wild type (Fig. 1F), which might explain the slightly lower magnetic response.

10.1128/mBio.00591-21.1 FIG S1 Simplified schematic illustration of an in-frame deletion of core-biosynthetic genes of *trans*-AT PKS cluster in Magnetospirillum gryphiswaldense, yielding the Δ*trans-at-pks* strain. Download FIG S1, TIF file, 0.4 MB. Copyright © 2021 Awal et al. 2021 Awal et al. https://creativecommons.org/licenses/by/4.0/ This content is distributed under the terms of the Creative Commons Attribution 4.0 International license.

To identify the biosynthetic product(s) of the *trans*-AT PKS cluster, the wild-type and Δ*trans-at-pks* strains were cultivated under aerobic, microaerobic, and anaerobic conditions in flask standard medium (FSM), and extracts of these strains were compared using principal-component analysis (Fig. S2) as previously described (32). Under microoxic and anoxic conditions, which are known to favor magnetosome biosynthesis (33, 34), there were no significant differences detectable between the mutant and the wild type. However, in the extract of the wild-type strain grown under aerobic conditions that are known to inhibit magnetosome formation (33, 34), we identified a compound with a mass of 691.38 Da which was absent from the Δ*trans-at-pks* mutant strain.

10.1128/mBio.00591-21.2 FIG S2 Principal-component analysis of the extracts of the M. gryphiswaldense wild type and Δ*trans-at-pks* strain. The more a component is responsible for a difference between the data sets, the farther it is to the right (wild type) or to the left (Δ*trans-at-pks*) from the center; the highlighted feature represents the target mass 692.38 *m/z*, which was assigned to the *trans*-AT PKS cluster and was found only in the wild-type strain. Download FIG S2, TIF file, 2.6 MB. Copyright © 2021 Awal et al. 2021 Awal et al. https://creativecommons.org/licenses/by/4.0/ This content is distributed under the terms of the Creative Commons Attribution 4.0 International license.

Yields of the target compound obtained from wild-type cultures proved insufficient for the isolation and subsequent elucidation of its structure by nuclear magnetic resonance spectroscopy (NMR). Since we hypothesized that the low production might be due to poor expression of biosynthetic genes, we attempted to enhance their expression by transcriptional activation. To this end, a DNA fragment of 145 bp harboring a putative native promoter in front of MSR-1_15600 (ORF7) was replaced with a 64-bp fragment containing the stronger constitutive promoter P*_mamDC_*_45_ (35) and the optimized ribosomal binding site (oRBS), yielding the *P_mamDC_*_45_*-trans-at-pks* strain (Fig. S3). P*_mamDC_*_45_ is an optimized version of the native promoter P*_mamDC_*, which drives transcription of the *mamGFDC* operon involved in magnetosome biosynthesis of M. gryphiswaldense (36), and was shown to enhance the expression of a foreign gene 8-fold compared to that obtained with P*_mamDC_* (35).

10.1128/mBio.00591-21.3 FIG S3 Simplified schematic illustration of the insertion of promoter P*_mamDC45_* in front of MSR-1_15600 in the *trans*-AT PKS cluster in Magnetospirillum gryphiswaldense, generating the *P_mamDC45_-trans-at-pks* strain. Download FIG S3, TIF file, 0.5 MB. Copyright © 2021 Awal et al. 2021 Awal et al. https://creativecommons.org/licenses/by/4.0/ This content is distributed under the terms of the Creative Commons Attribution 4.0 International license.

**FIG 2:**
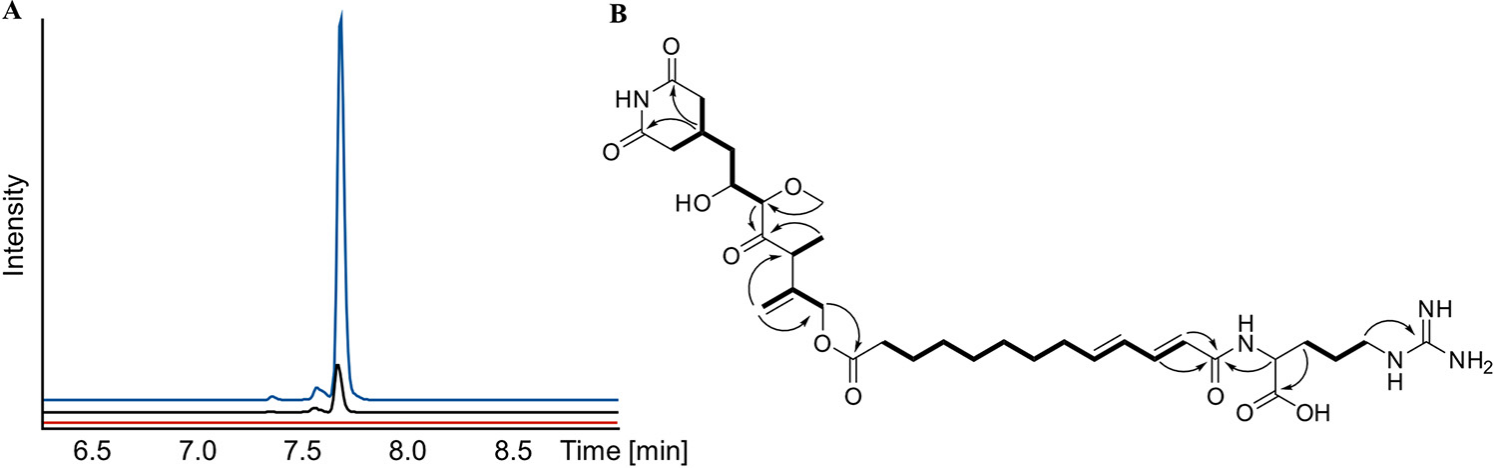
(A) Extracted ion chromatograms for *m/z* 692.38 [M+H]^+^ showing the difference in compound production. In the Δ*trans-at-pks* strain (red), the production of the compound was abolished. In the promoter-activated *P_mamDC45_-trans-at-pks* strain (blue), the production was increased ca. 7-fold (area under the curve [AUC], 6,513,288) in comparison to that of the wild type (black) (AUC, 880064). (B) NMR-elucidated structure of sesbanimide R with the most relevant COSY (bold) and HMBC (arrows) correlations.

Indeed, mass spectra obtained by liquid chromatography-mass spectrometry (LC-MS) from extracts of the *P_mamDC_*_45_*-trans-at-pks* strain showed a 7-fold-increased intensity of the target mass, suggesting a successful transcriptional activation of the gene cluster (Fig. 2A). As the yield of the compound obtained from shake flasks cultures of the *P_mamDC_*_45_*-trans-at-pks* strain was still too low for the isolation of the corresponding natural product, we scaled its production up to a 10-liter fermentor, which provided enhanced aeration and growth of the culture. This approach enabled the isolation of 2 mg of the compound by semipreparative high-performance liquid chromatography (HPLC) and the elucidation of its structure using MS and NMR spectrometry.

### *De novo* structure elucidation.

High-resolution electrospray ionization mass spectrometry (HRESI-MS) analysis of the compound (Fig. 2B) shows an [M+H]^+^ signal at *m/z* 692.3869 (calc. 692.3865 Δ = −0.6 ppm) consistent with the neutral sum formula of C_34_H_53_N_5_O_10_ and containing 11 double bond equivalents (DBEs). The ^1^H NMR and heteronuclear single quantum coherence (HSQC) spectra of the compound (Table 1) revealed a signal characteristic for an aliphatic exo double bond at *δ*(^1^H) of 5.18 and 4.96 ppm (H-13), which shows heteronuclear multiple bond correlation (HMBC) correlations to a methylene group at *δ*(^1^H) of 4.66 displayed as a singlet (H-10), as well as a methine group at *δ*(^1^H) of 3.71 ppm (H-8). The quartet of the methine group shows correlation spectroscopy (COSY) correlations to one methyl group at *δ*(^1^H) of 1.19 ppm (H-12). This methyl group exhibits HMBC correlations to the quaternary carbon participating in the exo double bond at *δ*(^13^C) of 144.5 ppm (C-9) besides a ketone at 213.2 ppm (C-7), indicating location of the methyl group and the methine group between the ketone (C-7) and the exo double bond (C-9 and C-13). On the other side of the ketone, another methine group with a chemical shift of *δ*(^1^H) of 3.66 ppm (H-6) is found, which is indicated by the HMBC correlations of those two groups. The moderately deshielded shift of this methine group in line with HMBC correlations to a methyl group with a moderately deshielded shift of *δ*(^1^H) of 3.40 ppm (H-11) reveals this part as a methoxy function. The methine group at *δ*(^1^H) of 3.66 ppm (H-6) furthermore shows COSY correlations to a second methine group at *δ*(^1^H) of 3.98 ppm (H-5). Its deshielded chemical shift suggests hydroxylation of this methine. It shows COSY correlations to a diastereotopic methylene group at *δ*(^1^H) of 1.49 and 1.73 ppm (H-4), which is located next to a methine group at 2.34 ppm (H-3) based on their COSY correlations. This methine group exhibits further COSY correlations to two diastereotopic methylene groups at *δ*(^1^H) of 2.36 and 2.68 (H-2a) and of 2.33 and 2.70 ppm (H-2b) with almost identical chemical shifts, wherefore they have to be located in almost identical chemical surroundings. They do not reveal any further COSY correlations but do reveal HMBC correlations to two quaternary carbons and at *δ*(^13^C) of 174.6 ppm (C-1a/b), which also show correlations to the methine group. Based on the sum formula of the molecule and the two-dimensional (2D) NMR data, the methine, the two methylenes, and the two quaternary carbons therefore likely are arranged as glutarimide, with substitution in position 4. There are no further correlations of any glutarimide participating functional groups; as a result, this part depicts one end of the molecule.

**TABLE 1:** NMR spectroscopic data for sesbanimide R in methanol-d_4_ at 500/125 MHz

No.	*δ* ^13^C (ppm)	*δ* ^1^H (ppm), mult (*J* [Hz])	COSY	HMBC
1a	174.6			
1b	174.6			
2a	39.1	2.36, 2.68, m	2b, 3	1a, 3, 4
2b	37.7	2.33, 2.70, m	2a, 3	1b, 3, 4
3	28.4	2.34, m	2a, 2b, 4	1a, 1b, 2a, 2b, 4, 5
4	38.9	1.49, 1.73, m	3, 5	2a, 2b, 3, 5, 6
5	71.7	3.98, dt (10.4, 3.1)	4, 6	3, 4, 6, 7
6	90.6	3.66, d (3.1)	5	4, 5, 7, 11
7	213.2			
8	46.9	3.71, q (7.0)	12	7, 9, 10, 12, 13
9	144.5			
10	66.8	4.66, s	13	8, 9, 13, 14
11	60.6	3.40, s		6
12	16.6	1.19, d (7.0)	8	7, 8, 9, 13
13	113.9	4.96, 5.18, s	10	8, 9, 10
14	174.6			
15	34.8	2.38, t (7.5)	16	14, 16, 17
16	26.0	1.63, m	15, 17	14, 15, 17, 18
17	30.4	1.34, m	16, 18	15, 16, 18, 19
18	30.1	1.34, m	17, 19	16, 17, 19, 20
19	30.6	1.30, m	18, 20	17, 18, 20, 21
20	29.7	1.45, m	19, 21	18, 19, 21, 22
21	33.8	2.18, q (7.1)	20, 22	19, 20, 22, 23
22	144.1	6.10, dt (7.2, 15.1)	21, 23	20, 21, 23, 24, 25
23	129.7	6.22, dd (10.8, 15.1)	22, 24	21, 22, 24, 25
24	142.3	7.12, dd (10.7, 15.1)	23, 25	22, 23, 25, 26
25	122.9	6.02, d (15.1)	24	22, 23, 24, 26
26	168.3			
27	177.6			
28	54.9	4.40, dd (5.3, 7.7)	nd	26, 27, 29, 30
29	30.9	1.74, 1.92, m	30	27, 28, 30, 31
30	26.0	1.63, m	29, 31	28, 29, 31
31	41.9	3.22, m	30	29, 30, 32
32	158.4			

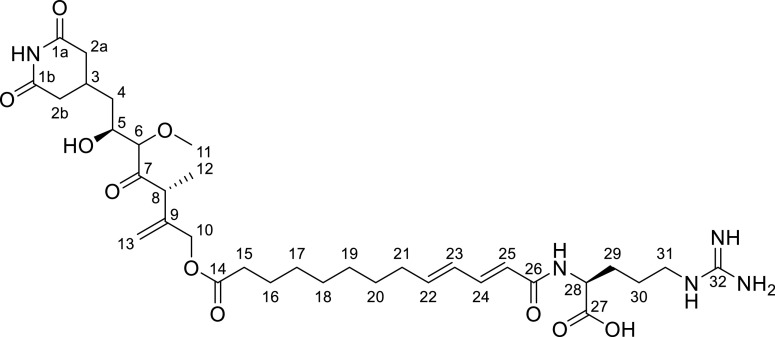

**FIG 3:**
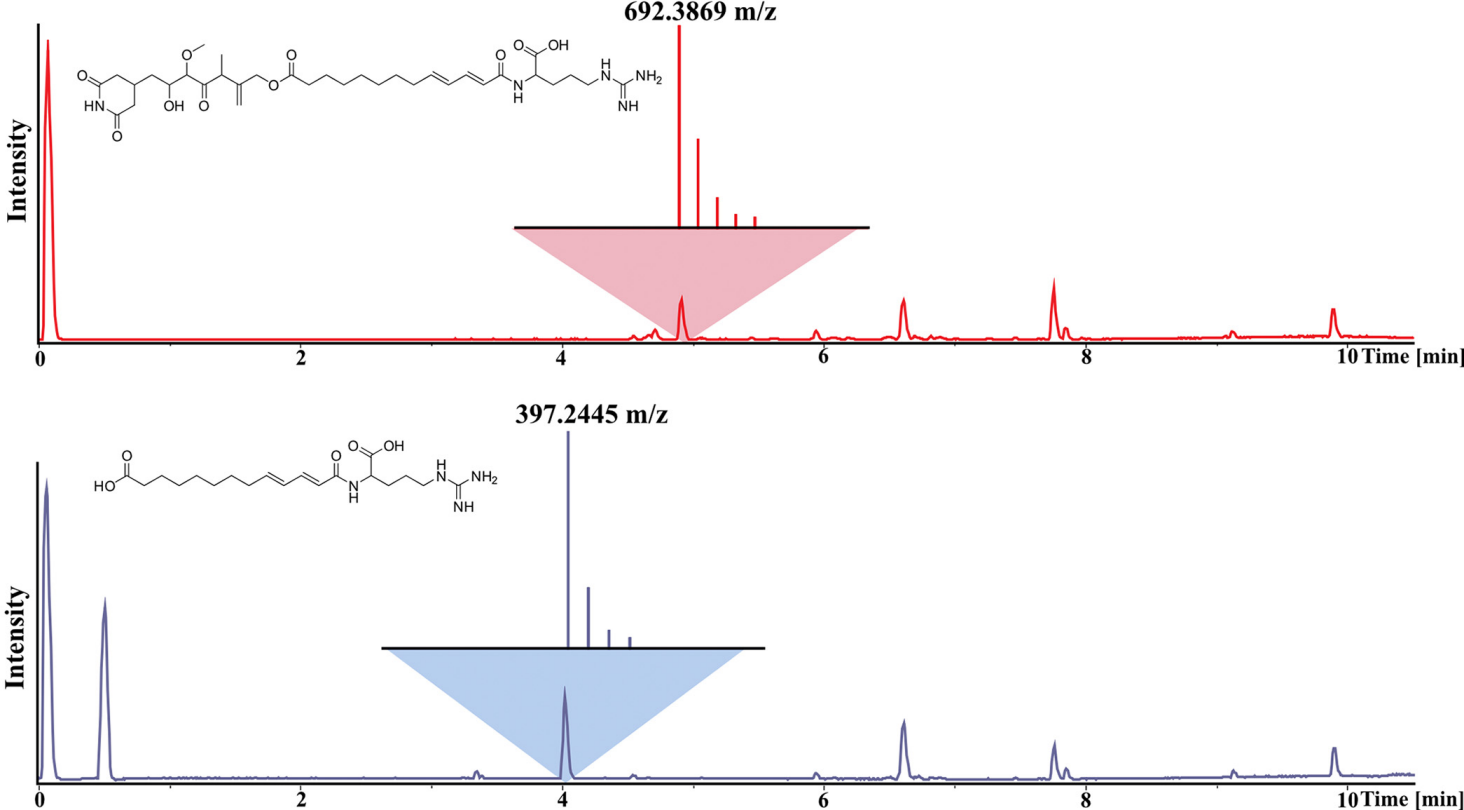
Saponification of sesbanimide R to confirm the NMR-elucidated structure. Shown are base peak chromatograms of a sesbanimide R sample before (top) and after (bottom) treatment with NaOH. A fragment with an *m/z* of 397.2445 [M+H]^+^ was observed, which corresponds to the arginine-containing part of the molecule after ester hydrolysis.

Besides correlations of the H-10 methylene to the partial structure described above, it shows HMBC correlations to a quaternary carbon at *δ*(^13^C) of 174.6 ppm (C-14). The deshielded chemical shift of this quaternary carbon suggests an ester bond in this position, which was confirmed by a saponification reaction (Fig. 3). The following seven methylene groups are arranged in a straight aliphatic chain, based on their chemical shifts and COSY as well as HMBC correlations. The deshielded chemical shift of the last of these seven methylene groups at *δ*(^1^H) of 2.18 ppm (H-21) and its signals displayed as quartet suggest that it is followed by the first of four dienone double bond protons at *δ*(^1^H) of 6.10, 6.22, 7.12, and 6.02 ppm (H-22-25). The two double bonds are conjugated based on COSY correlations of the four dienone double bond protons and their deshielded carbon chemical shifts at *δ*(^13^C) of 144.1, 129.7, 142.3, and 122.9 ppm (C-22-25). The last two double bond protons at *δ*(^1^H) of 7.12 and 6.02 ppm (H-24 and H-25) show HMBC correlations to a quaternary carbon at *δ*(^13^C) of 168.3 ppm (H-26). Its characteristic chemical shift and additional correlations of an alpha proton (H-28) to this quaternary carbon (C-26) reveal it as acid function of an amide bond. The alpha proton at *δ*(^1^H) of 4.40 ppm (H-28) belongs to arginine, which was confirmed by Marfey’s analysis (59) in addition to the following NMR correlations. It shows HMBC correlations to a free carboxylic acid function at *δ*(^13^C) of 177.6 ppm (C-27) and two methylene groups and at *δ*(^1^H) of 1.92 and 1.63 ppm (H-29 and H-30), which themselves show HMBC correlations to a third more moderately deshielded chemically shifted methylene group at *δ*(^1^H) of 3.22 ppm (H-31). Its characteristic chemical shift and correlations to a quaternary carbon at *δ*(^13^C) of 158.4 ppm (C-32) reveal the coupling to the guanidine moiety of the molecule here, which marks the other end of compound. In addition to the NMR data, we observed a fragment of *m/z* 397.245 [M+H]^+^ with LC-MS after saponification of the ester (Fig. 3). The size and sum formula of this fragment correspond to the arginine plus aliphatic chain containing part of the molecule and confirms the elucidated structure. This structure is highly similar to the structure of sesbanimide F, which became available at a late stage of our work in a study by Kačar et al. (28, 60). However, our compound contains an additional terminal arginine (R) moiety. Hence, we used the name sesbanimide R.

### Determination of the sesbanimide R stereochemistry.

The vicinal coupling constant of 15.1 Hz for both aliphatic double bonds suggests an *E*-configuration of both double bonds. Marfey’s analysis and comparison to commercially available l- and d-arginine standards revealed the arginine from sesbanimide R to be *S* configured, as the hydrolysis product of sesbanimide R has the same retention times as l-arginine when derivatized with fluorodinitro-phenyl-5-l-leucine amide (l-FDLA) and d-FDLA, respectively (Table 2). Due to instabilities of the molecule under acidic and basic conditions and selectivity issues between the free hydroxyl groups and the glutarimide, Mosher esterification experiments, which were carried out to elucidate the configuration of the remaining stereocenters, were not successful. When adding 10 or fewer equivalents of pyridine to the reaction mixture in chloroform, we observed complete degradation of the molecule. When performing the experiment with pure pyridine, the hydroxyl group underwent fast elimination after formation of the respective Mosher ester. We were therefore not able to determine the absolute stereochemical configuration of the molecule experimentally and speculate on the stereochemistry based on *in silico* analysis of the BGC. The *trans*ATor tool predicts the structure of *trans*-AT polyketides according to the substrate specificities of the involved keto-synthase (KS) domains (37). The top five hits of the tool predict the KS domain of module 4 to accept d-OH, while the sequence-based stereochemistry prediction for the ketoreductase (KR) domain of module 3 was inconclusive. We therefore predict the stereocenter at C-5 to be *S* configured. Xie et al. (38) recently suggested that all C-methyltransferases in *trans*-AT PKS assembly lines generate (2*R*)-2-methyl-3-ketoacyl-acyl carrier protein (3-ketoacyl-ACP) intermediates and that (2*S*)-2-methyl-3-hydroxyacyl-ACP intermediates are produced by epimerizing A2-type KR domains (38). As there is no KR domain present in module 4, we propose that the stereocenter at C-8 is *R* configured. The stereocenter at C-6 is likely generated by a cytochrome P450 (cyP450) enzyme (SbnE), but we were not able to make a prediction for its stereochemistry. These predictions are speculative, and further experiments are required to fully elucidate the stereochemistry of sesbanimide R.

**TABLE 2:** Retention times for l- and d-arginine standards as well as sesbanimide R after derivatization with l- and d-FDLA to determine the stereochemistry of the arginine moiety of sesbanimide R

Sample	Retention time (min)		Assignment
	d-FDLA	l-FDLA	
d-Arginine	10.85	10.35	
l-Arginine	10.35	10.85	
Hydrolysis of sesbanimide R	10.88	10.36	*S* configured

### *In silico* analysis of the gene cluster and biosynthesis hypothesis.

**FIG 4:**
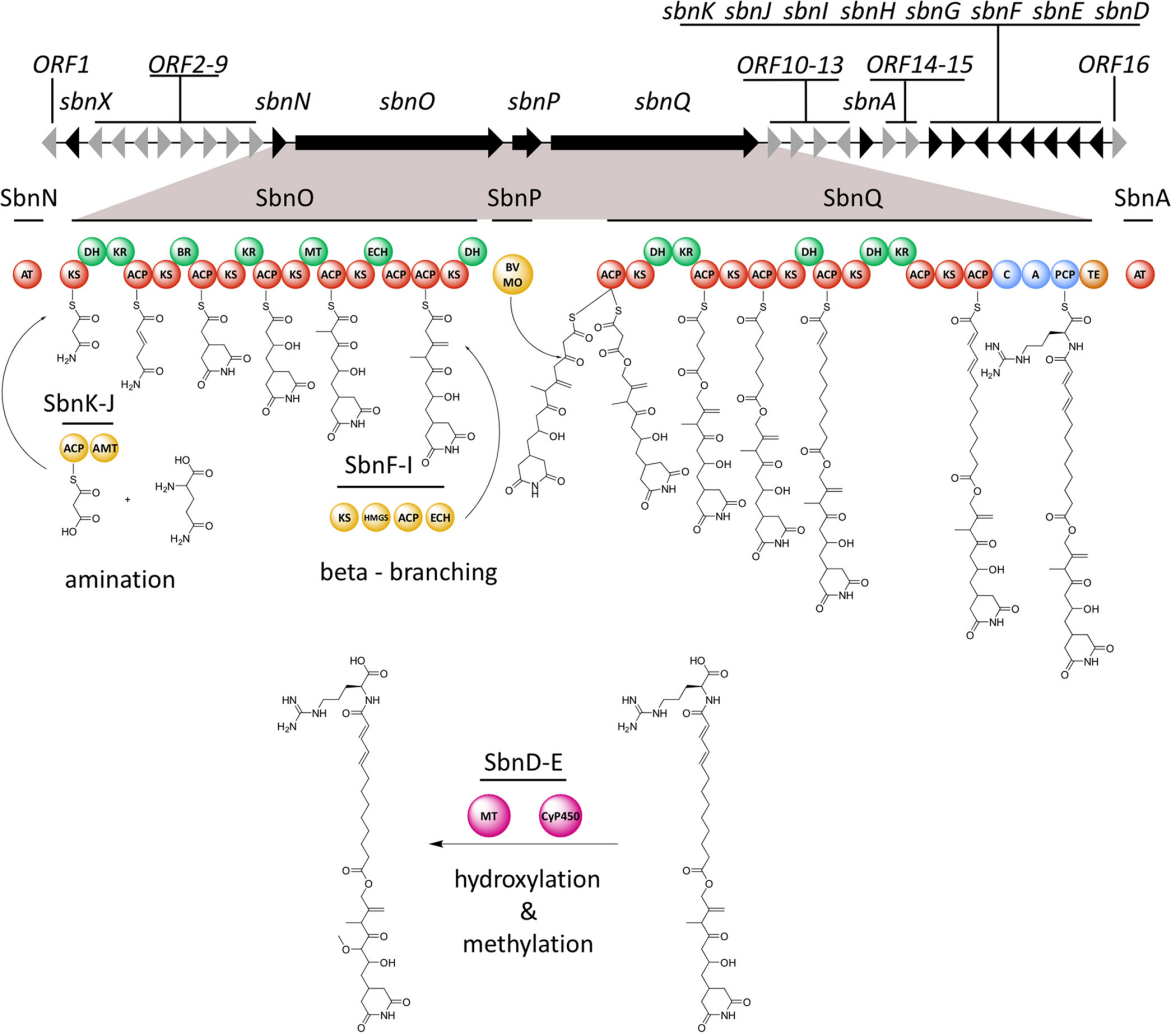
Proposed biosynthetic pathway for sesbanimide R. Core PKS modules are marked in red, core NRPS modules are marked in blue, and the thioesterase is marked in orange. DH and KR modules of the core assembly line are marked in green. The amidotransferase, beta-branching cassette, and Baeyer-Villiger monooxygenase are marked in yellow and the tailoring methyltransferase and cyP450 enzyme in pink. The genes involved in the sesbanimide R biosynthesis are marked in black and named sbnA-X. The remaining genes with unknown or unassigned functioned are marked in gray and named ORF 1 to 16.

A detailed annotation of the BGC was carried out (Table S2). Besides using antiSMASH (30) for cluster and domain identification, additional information was gained by submitting the translated protein sequences to the *trans*ATor tool (37) (Table S3). Finally, the conserved domain search tool CDD (58) was used to identify domains that were not identified by antiSMASH. The core biosynthetic gene cluster (BGC) spans over 39 kbp and consists of the two large PKS genes, *sbnO* (MSR-1_15630) and *sbnQ* (MSR-1_15650), as well as one monooxygenase-encoding gene, *sbnP* (MSR-1_15640). The core cluster is flanked by two acyltransferase (AT) domains encoded by *sbnA* and *sbnN*. SbnN was identified as an *in-trans* acyltransferase and SbnA as an *in-trans* acylhydrolase. Several additional biosynthetic genes are encoded up- and downstream of the core BGC: an asparagine synthase accompanied by an ACP domain (*sbnJ* and *sbnK*), a beta-branching cassette (39) (*sbnF*-*I*), a cytochrome P450 enzyme (*sbnE*), a methyltransferase (*sbnD*), and a stand-alone acyl-coenzyme A (acyl-CoA) dehydrogenase (s*bnX*). ORFs 6, 8, 9, 11, 14, and 15 encode transport-associated proteins putatively responsible for exporting sesbanimide R out of the cell. ORFs 2, 3, 5, and 7 encode regulatory proteins putatively responsible for controlling BGC expression and thereby sesbanimide production. ORFs 4, 10, 12, and 13 were annotated as encoding hypothetical proteins with unknown function. All KS domains of SbnO and SbnQ possess the active-site cysteine and histidine, except for the nonelongating KS5 of SbnQ, which is missing the first histidine (40). All ACP domains of SbnO and SbnQ possess the canonical active-site serine. The catalytic triad of serine, tyrosine, and asparagine is present in all KR domains of SbnO and SbnQ. The dehydratase (DH1) of SbnO and DH1 and DH2 of SbnQ contain the conserved HXXXGXXXXP motive, which is missing in DH2 of SbnO and DH3 of SbnQ (Fig. S4A to D). We therefore propose the following biosynthesis scheme, based on *in silico* analysis of the BGC and considering the elucidated chemical structure of sesbanimide R (Fig. 4).

10.1128/mBio.00591-21.4 FIG S4 (A) Alignment of ACP domains of *sbnO* and *sbnQ*. All ACPs contain the active-site serine. (B) Alignment of KS domains of *sbnO* and *sbnQ*. All KS domains contain the conserved cysteine and two histidines except for KS5 of *sbnQ*. (C) Alignment of DH domains of *sbnO* and *sbnQ*. DH2 (DHt.1) of *sbnO* and DH3 (DHt.1) of *sbnQ* are missing the conserved HXXXGXXXXP motif. (D) Alignment of KR domains of *sbnO* and *sbnQ*. All domains contain the conserved active-site residues serine, tyrosine, and asparagine. Download FIG S4, TIF file, 3.0 MB. Copyright © 2021 Awal et al. 2021 Awal et al. https://creativecommons.org/licenses/by/4.0/ This content is distributed under the terms of the Creative Commons Attribution 4.0 International license.

10.1128/mBio.00591-21.7 TABLE S2 *In silico* analysis of the sesbanimide biosynthetic gene cluster from Magnetospirillum gryphiswaldense. Annotation of the sesbanimide gene cluster in Magnetospirillum gryphiswaldense and comparison the sesbanimide cluster in PHM037 and PHM038. Download Table S2, DOCX file, 0.01 MB. Copyright © 2021 Awal et al. 2021 Awal et al. https://creativecommons.org/licenses/by/4.0/ This content is distributed under the terms of the Creative Commons Attribution 4.0 International license.

10.1128/mBio.00591-21.8 TABLE S3 Substrate specificities as predicted by the *trans*ATor tool and sorted according to E value. The predictions that fit the structure and biosynthesis proposal are highlighted in bold. Download Table S3, DOCX file, 0.01 MB. Copyright © 2021 Awal et al. 2021 Awal et al. https://creativecommons.org/licenses/by/4.0/ This content is distributed under the terms of the Creative Commons Attribution 4.0 International license.

Initially, an amino group is transferred to ACP bound malonate by SbnJ (41). The starter moiety is then transferred to the first module of SbnO. Modules one and two of the assembly line then form the glutarimide moiety as previously described for the biosynthesis of gladiofungin (42). Modules three to five elongate the nascent molecule according to the substrate specificity prediction for their KS domains. Exomethylene moiety incorporation by module five has previously been described for several *trans*-AT PKS biosyntheses (43). The domains required for exomethylene formation (ECH domain, tandem ACP domains, and a beta-branching cassette [*sbnF*-*I*]) are all present in the cluster. Module six is found split onto the genes *sbnO* and *sbnQ*, which are separated by *sbnP*, encoding a flavin-binding monooxygenase. Such a split module, containing a monooxygenase and a DH domain missing the conserved HXXXGXXXXP motif, has been shown to incorporate oxygen into polyketide backbones. The monooxygenase accepts thioesters bearing β-keto groups and acts as a Baeyer-Villiger monooxygenase (BVMO) to generate malonyl esters (29). The module is thus likely responsible for the ester formation in sesbanimide R. The second part of the molecule is synthesized by the PKS megasynthase SbnQ. Judging by the structure formula of sesbanimide R, we propose that module seven or eight performs one iterative PKS elongation step and thus incorporates a second malonyl-CoA building block into the final molecule. The DH and KR domains are proposed to act in *trans*, to biosynthesize the saturated part and double bonds present in sesbanimide R. An enoylreductase (ER) domain would also be needed to fully reduce the incorporated C-2 unit, but this domain is not encoded on *sbnQ*. We therefore propose that this function is carried out by SbnX, which was identified as an acyl-CoA dehydrogenase. The terminal NRPS module on *sbnQ* was predicted to incorporate l-arginine by NRPS predictor 2, which fits well with the elucidated structure (44). We propose that the methoxy group at C6 is incorporated by a cytochrome P450 enzyme and an Fkbm family methyltransferase, encoded by *sbnE* and *sbnD*, respectively.

Taken together, our devised biosynthesis scheme for sesbanimide R is very similar to the pathway suggested in parallel by Kačar et al. for the biosynthesis of sesbanimide F from Stappia indica PHM037 (28, 60). The main differences between the two BGC lie in the distribution of DH and KR domains in SbnQ, the presence of three additional transport-associated genes in the M. gryphiswaldense cluster, and a phosphopantetheinyl transferase in the Stappia indica cluster which is absent in the M. gryphiswaldense BGC. Notably, the final products from the strains under investigation by Kačar et al. do not contain the terminal arginine moiety observed in sesbanimide R, even though the corresponding biosynthetic gene cluster contains the l-arginine-incorporating NRPS module (28, 60). We speculate that the BGC from M. gryphiswaldense responsible for sesbanimide R formation is an evolutionary intermediate in a developmental line leading to the sesbanimide gene cluster from PHM037 and PHM038 or that these clusters may carry a nonfunctional NRPS module. A conserved domain search of the adenylation (A) domain of the NRPS modules from M. gryphiswaldense and *S. indica* PHM037 revealed that the active sites are likely intact in both cases. In the case of the *S. indica* domain, however, the residues just before the active site seem to be unusual for A-domains. They were identified because they do not match the alignment against the reference A-domains from the CDD database (58) (Fig. S5A and B). Kačar et al. speculated that the arginine moiety is cleaved rapidly after the biosynthesis, so that the corresponding analogues are not detectable with the applied analytical conditions (28, 60). As we were able to detect sesbanimide R, which was also relatively stable, we suggest as an alternative explanation that the uncommon residues close to the active-site residues might result in an inactive A-domain in the *S. indica* cluster and that, therefore, no arginine is incorporated. Additionally, we did not detect any of the sesbanimides (A, B, C, D, E, and F) which were observed by Kačar et al. (28, 60) in M. gryphiswaldense. A possible explanation might be that the tailoring steps resulting in the formation of sesbanimides A, D, C, and E occur only if no arginine moiety is present. However, until further insight is gained into the biosynthesis of these compounds, the reason(s) for the discrepancy in product composition remains elusive.

10.1128/mBio.00591-21.5 FIG S5 (A) Alignment of the amino acid sequence of the A-domain from the last module of SbnQ from *S. indica* PHM037 with the reference domains of the CDD search tool. The active-site residues are highlighted in yellow. The residues just before the active site (red box) do not match the reference sequences in length. (B) Alignment of the amino acid sequence of the A-domain from the last module of SbnQ from M. gryphiswaldense with the reference domains of the CDD search tool. The active-site residues are highlighted in yellow. Download FIG S5, TIF file, 2.8 MB. Copyright © 2021 Awal et al. 2021 Awal et al. https://creativecommons.org/licenses/by/4.0/ This content is distributed under the terms of the Creative Commons Attribution 4.0 International license.

### Cytotoxicity.

Sesbanimides have been associated with strong antitumor/cytotoxic activities, which is common for polyketides containing a glutarimide moiety (45, 46). We therefore tested sesbanimide R *in vitro* against cell lines of liver carcinoma (HepG2), endocervical adenocarcinoma (KB3.1), colon carcinoma (HCT-116), and lung carcinoma (A549). The 50% inhibitory concentration (IC_50_) values against HePG2 (23 nM; 95% confidence interval [CI], 9 to 65), HCT-116 (39 nM; 95% CI, 28 to 54), and KB3.1 (20 nM; 95% CI, 15 to 28) are comparable to those of sesbanimide F, which exhibits a compound concentration that produces 50% cell growth inhibition (GI_50_) of 20 nM against A549 cells (28, 60). To better compare sesbanimides R and F, we also tested sesbanimide R against A549 cells, which resulted in an IC_50_ of 30 nM (95% CI, 21 to 40). These results indicate that the arginine moiety has no effect on cytotoxicity, which falls well within the range commonly observed for glutarimide-containing polyketides and other members of the sesbanimide compound family (28, 46, 47, 60).

### Conclusion.

We unambiguously assigned a new member of the sesbanimide compound family to a *trans-*AT polyketide synthase biosynthetic gene cluster from Magnetospirillum gryphiswaldense by inactivation and overexpression of the cluster and statistical analysis of the strains’ metabolome.

Sesbanimide R belongs to the sesbanimide family of natural products. We suggest a biosynthesis pathway which is largely in line with the one proposed in a parallel study for sesbanimides A, C, D, E, and F (28, 60). In contrast to these compounds, sesbanimide R contains a terminal arginine moiety, which perfectly matches the *in silico* predictions of the BGC.

Sesbanimides were isolated originally from the seeds of Sesbania drummondii (48) and later from marine agrobacteria, indicating that symbiotic microorganisms are the actual sources for these metabolites rather than the plant (28, 49, 60), a finding which is further supported by our study. Sesbanimide R is of interest owing to its cytotoxic bioactivity against several carcinoma cell lines, which is a characteristic of glutarimide-containing polyketides (45, 46). The potent cytotoxic activity makes it a candidate for further investigations regarding its mode of action and development as an antitumor agent. As in other bacteria, the role of sesbanimide R for the physiology and fitness of M. gryphiswaldense in its freshwater habitat remains elusive and requires further investigations.

Sesbanimide R is the first natural product identified and isolated from a magnetotactic bacterium. In addition to its well-established property to produce biogenic magnetic nanoparticles, it makes the tractable strain M. gryphiswaldense highly interesting also as a producer of secondary metabolites. Since numerous biosynthetic gene clusters encoding putative polyketide synthases and nonribosomal peptide synthetases are present in the genomes of many different MTB (Table S4), our study sets the stage for exploring this highly diverse group of prokaryotes as a potential source for the future discovery of novel secondary metabolites.

10.1128/mBio.00591-21.9 TABLE S4 Putative open reading frames (ORFs) encoding polyketide synthases (PKSs), nonribosomal peptide synthetases (NRPSs), or hybrid in PKS and NRPS gene clusters from different magnetotactic bacteria. *, genome assembly of these species is not complete, and the number of ORFs might be variable in the final analysis. Download Table S4, DOCX file, 0.02 MB. Copyright © 2021 Awal et al. 2021 Awal et al. https://creativecommons.org/licenses/by/4.0/ This content is distributed under the terms of the Creative Commons Attribution 4.0 International license.

## MATERIALS AND METHODS

### *In silico* analysis of the genome of magnetotactic bacteria and bioinformatics methods.

The Magnetospirillum gryphiswaldense genome (accession no. CP027527) and genomes of other magnetotactic bacteria were screened for secondary metabolite biosynthetic gene clusters using the bioinformatic tool antiSMASH (version 5.1.2) (30, 57). The amino acid sequence was aligned with the Basic Local Alignment Search Tool (BLASTp) against the publicly available database to find homologous proteins and to predict the functions of the ORFs. The presence of homologous ORFs in PHM037/PHM038 strains (28, 60) was searched using the software Geneious Prime (Biomatters Ltd., Auckland, New Zealand; 2020.0.3). Furthermore, PKS and NRPS domain architecture and specificities present in the cluster were considered using TransAT (http://transator.ethz.ch) or Pfam database (50).

### Bacterial strains and culture conditions.

Escherichia coli was grown in lysogeny broth (LB) at 37°C and shaking at 180 rpm. Donor strain E. coli WM3064 (W. Metcalf, unpublished data) was cultivated with 0.1 mM dl-α,Ɛ-diaminopimelic acid (DAP). M. gryphiswaldense was grown microaerobically at 28°C in modified flask standard medium (FSM) (33) with moderate agitation at 120 rpm, if not mentioned otherwise. Optical density (OD) and magnetic response (*C*_mag_) of M. gryphiswaldense strains were determined photometrically at 565 nm as reported earlier (31). Antibiotic selection was achieved by the addition of kanamycin at concentrations of 5 μg/ml (M. gryphiswaldense) and 25 μg/ml (E. coli). For agar media, 1.5% (wt/vol) agar was added to the liquid culture medium. Strains and vectors used in this study are shown in Table S5.

10.1128/mBio.00591-21.10 TABLE S5 Strains, vectors, and primers used in this study. Download Table S5, DOCX file, 0.03 MB. Copyright © 2021 Awal et al. 2021 Awal et al. https://creativecommons.org/licenses/by/4.0/ This content is distributed under the terms of the Creative Commons Attribution 4.0 International license.

### Molecular and genetic techniques.

Oligonucleotides (Table S5) were purchased from Sigma-Aldrich (Steinheim, Germany). Chromosomal DNA of M. gryphiswaldense was isolated using a kit from Zymo Research, USA. Plasmids were constructed by standard recombinant techniques as described below. All constructs and selected amplicons from the mutants were sequenced by Macrogen Europe (Amsterdam, Netherlands).

### Construction of markerless site-specific deletion and activation of *trans*-AT PKS cluster.

Markerless in-frame deletion of core-biosynthetic biosynthetic genes of the *trans*-AT PKS cluster and insertion of a promoter in front of the cluster were conducted using homologous recombination based on counterselection systems described previously (51). For the construction of the deletion plasmid, homologous regions of ca. 1.6 kb located upstream of MSR-1_15620 (locus tag) including the first three codons of MSR-1_15620 and downstream of MSR-1_15650 with its last three codons were amplified from genomic DNA (gDNA) of M. gryphiswaldense using a proofreading DNA polymerase and primer pairs RPA595/RPA596 and RPA597/RPA598. The PCR products were purified from the agarose gel using a gel extraction kit (Zymo Research, USA) and cloned into pORFM (51) digested with SalI and NotI by Gibson assembly (52).

For activation of the *trans*-AT PKS cluster, the strong promoter P*_mamDC_*_45_ with the spacing-optimized ribosome binding site (oRBS) was amplified from pAP150 (35) with primer pair RPA939/940. Homologous arms consisting of ca. 1.5 kb of the C terminus of MSR1_15590 and N terminus of MSR1_15600 were amplified from gDNA of M. gryphiswaldense using primer pairs RPA937/938 and RPA940/941. The purified PCR products were assembled into pORFM (51) digested with SalI and NotI by Gibson assembly (52) with the P*_mamDC45_*-oRBS in between the two homologous arms. Five microliters of the Gibson assembly reaction was transformed into chemically competent E. coli DH5α (53), and the presence of the cloned fragment was confirmed by colony PCR using pair RPA484/485. The plasmid was isolated from the correct clone using a Zymo Research kit and sequenced by Macrogen Europe (Amsterdam, Netherlands).

### Conjugation.

Plasmid transfer by biparental conjugation was performed with donor strain E. coli WM3064 consisting of the verified construct and M. gryphiswaldense as the acceptor strain as reported previously (25). In-frame markerless chromosomal deletion and insertion were generated following the conjugative transfer of the plasmid to M. gryphiswaldense and homologous recombination utilizing GalK-based counterselection as previously described (51). Successful deletion and insertion yielded Δ*trans-at-pks* and *P_mamDC45_-trans-at-pks* strains, respectively. The mutants were confirmed by PCR using primers (Table S5) specific to sequences adjacent to the homologous regions and were verified by Sanger sequencing of the amplicons.

### Growth curve and cell length analyses.

For growth analyses, the strains were grown in 24-well plates (Sarstedt, Nümbrecht, Germany) in 1 ml of FSM (33) in a microplate reader (Infinite 200 PRO; Tecan, Switzerland) with an automated reading of absorbance (560 nm) every 20 min for 150 cycles under aerobic conditions at 28°C with shaking at 140 rpm. Absorbance values were corrected using FSM as a blank. Cell length of the strains was estimated with the ImageJ plugin MicrobeJ 5.13i (54) using the SHAPElength cell shape descriptor. Analysis of cell length was done as reported previously (55).

### Transmission electron microscopy.

For TEM analysis, strains (wild type, Δ*trans-at-pks*, and *P_mamDC45_-trans-at-pks*) cultivated in 6-well plates (Sarstedt, Nümbrecht, Germany) under microoxic conditions at 24°C for 48 h, fixed in formaldehyde (1.8%), adsorbed onto carbon-coated copper grids (F200-CU carbon support film, 200 mesh; Electron Microscopy Sciences, Hatfield, UK), and washed three times with double-distilled water (ddH_2_O). TEM was performed on a JEM-2100 instrument (JEOL Ltd., Tokyo, Japan) with an accelerating voltage of 80 kV. Images were captured with a Gatan model 782 ES500W Erlangshen charge-coupled-device (CCD) camera (Gatan Inc., Pleasanton, CA) with the software Digital Micrograph 1.80.70 (Gatan Inc.). For data analysis and measurements, the software ImageJ Fiji V1.50c (56) was used.

### Cultivation of strains for statistical analysis of the metabolome.

For the screening of secondary metabolites, M. gryphiswaldense and Δ*trans-at-pks* strains were cultivated at 28°C in FSM (33) with an initial OD at 565 nm (OD_565_) of 0.01 under aerobic, microoxic, and anaerobic conditions in 500-ml baffled Erlenmeyer flasks, in Duran Laboratory flasks with rubber stoppers containing 50 ml of medium, and in 250-ml Duran Laboratory flasks containing 240 ml of degassed medium with rubber stoppers, respectively. One milliliter (vol/vol) of sterile Amberlite resin XAD-16 (Sigma-Aldrich Chemie GmbH, Taufkirchen, Germany) was added to the culture grown under aerobic and microoxic conditions and 5 ml (vol/vol) of XAD-16 into the culture grown under anaerobic conditions. The culture under aerobic condition was agitated at 150 rpm. The cells and the resin were harvested together by centrifugation after 60 h of incubation before extraction.

To access the activation of the cluster, wild-type, *P_mamDC45_-trans-at-pks*, and Δ*trans-at-pks* strains were cultivated under aerobic conditions at 28°C in 100 ml of FSM in a 1-liter baffled Erlenmeyer flask with a starting OD_565_ of 0.01 at 150 rpm. The culture was supplemented with 2 ml (vol/vol) sterile Amberlite resin XAD-16 (Sigma-Aldrich Chemie GmbH, Taufkirchen, Germany). After 60 h of incubation, the cells and resin were harvested together by centrifugation.

### Fermentor cultivation.

Up-scale cultivation of the *P_mamDC45_-trans-at-pks* strain was done in a 10-liter BioFlow 320 fermentor (Eppendorf Bioprocess, Jülich, Germany) (34) under aerobic conditions with an initial OD_565_ of 0.04. A 900-ml culture grown under aerobic conditions was used as an inoculum for a 9-liter culture which was supplemented with 200 ml (vol/vol) of XAD-16. The cells and resin were harvested together by centrifugation after 60 h of incubation and dried in a lyophilizer before extraction.

### Extraction of the cell pellet and resin.

For screening of the target compound(s), the cell pellet and resin of each culture were extracted with 50 ml of methanol for 1 h. The extract was then dried and resolved in 2 ml of methanol. This extract was then centrifuged for 5 min at 215 × *g* and diluted 1:10 prior to analysis with LC-MS system 1a and processing with Metaboscape 5.0 (Bruker).

### Extraction and isolation of sesbanimide R.

The dry cells and resin from the upscaled fermentation were extracted three times with 500 ml of methanol. The extract was subsequently partitioned between hexane, ethyl acetate, and water. Sesbanimide R was detected solely in the aqueous layer. This layer was then dried and resuspended in methanol. Sesbanimide R was isolated from this prepurified extract using LC-MS system 2. During purification, it became apparent that sesbanimide R is unstable during prolonged exposure to light and oxygen simultaneously. Therefore, all purification steps were carried out with minimal exposure to light.

### LC-MS systems.

All analytical LC-MS measurements were performed on a Dionex Ultimate 3000 RSLC system using a BEH C_18_, 100- by 2.1 mm, 1.7-μm particle diameter (dp) column (Waters, Germany), coupled to a maXis 4G high-resolution time of flight (HR-ToF) mass spectrometer (Bruker Daltonics, Germany) using an Apollo electrospray ionization (ESI) source. UV spectra were recorded by a diode array detector (DAD) in the range of 200 to 600 nm. The LC flow was split to 75 μl/min before entering the mass spectrometer.

### LC-MS system 1a: standard measurements.

Separation of 1 μl of sample was achieved by a linear gradient from H_2_O plus 0.1% formic acid (FA) (solvent A) to acetonitrile (ACN) plus 0.1% FA (solvent B) at a flow rate of 600 μl/min and 45°C. The gradient was initiated by a 0.5-min isocratic step at 5% solvent B, followed by an increase to 95% solvent B in 18 min to end up with a 2-min step at 95% solvent B before reequilibration under the initial conditions. Mass spectra were acquired in centroid mode ranging from 150 to 2,500 *m/z* at a 2-Hz scan rate.

### LC-MS system 1b: Marfey’s method.

Separation of 1 μl of sample was achieved by a gradient from H_2_O plus 0.1% FA (solvent A) to ACN plus 0.1% FA (solvent B) at a flow rate of 600 μl/min and 45 °C. The gradient was as follows: ramp in 1 min from 5% solvent B to 10% solvent B, in 14 min to 35% solvent B, in 7 min to 55% solvent B, and in 3 min to 80% solvent B. This is followed by a 1-min step at 80% solvent B before reequilibration with the initial conditions. Mass spectra were acquired in centroid mode ranging from 250 to 3,000 *m/z* at a 2-Hz scan rate.

### LC-MS system 1c: MS/MS measurements.

Separation of 1 μl of sample was achieved by a linear gradient from H_2_O plus 0.1% FA (solvent A) to ACN plus 0.1% FA (solvent B) at a flow rate of 600 μl/min and 45°C. The gradient was initiated by a 0.5-min isocratic step at 5% solvent B, followed by an increase to 95% solvent B in 18 min to end up with a 2-min step at 95% solvent B before reequilibration under the initial conditions. Mass spectra were acquired in centroid mode ranging from 150 to 2,500 *m/z* at a 2-Hz scan rate. Ions were selected for fragmentation by scheduled precursor list, and the collision energy was determined by mass- and charge state-dependent stepping from 25 to 60 eV.

### LC-MS system 2.

The final purification was performed on a Dionex Ultimate 3000 SDLC low-pressure gradient system using a Luna 5-μm, C_18_(2), 100-Å, 250- by 100-mm column (Phenomenex). Separation of 50 μl of sample was achieved by a gradient from H_2_O plus 0.1% FA (solvent A) to ACN plus 0.1% FA (solvent B) at a flow rate of 5 ml/min and 45°C. The gradient was as follows: a 2-min isocratic step at 5% solvent B, followed by a ramp to 35% solvent B in 3 min, a ramp to 50% solvent B in 20 min, and a ramp to 95% solvent B in 1 min. The 3-min wash step was followed by a return to initial conditions in 1 min and reequilibration for 3 min. UV spectra were recorded by a DAD in the range of 200 to 600 nm. The LC flow was split to 0.525 ml/min before entering the Thermo Fisher Scientific ISQ EM single quadrupole mass spectrometer. Mass spectra were acquired by selected ion monitoring (SIM) at *m/z* 692.38 [M+H]^+^.

### Statistical analysis.

Duplicates of wild-type and Δ*trans-at-pks* cultures were measured twice with LC-MS system 1a. Feature finding and bucketing were performed with the following parameters: minimum intensity, 5,000; minimum spectra for extraction, 5; and minimum spectra for recursive feature extraction, 3. Recursive feature extraction was performed when a feature was present in 2 out of 8 analyses, and features were included in the bucket table when present in 3 out of 8 analyses after recursive feature extraction. Principal-component analysis (PCA) was performed to find differences between the two groups (wild type and Δ*trans-at-pks*), with four analyses in each group. The PCA results were normalized with a logarithmic algorithm to account for low-intensity features. Features that accounted for the largest difference between the data sets were reevaluated in the raw data.

### Structure elucidation by NMR.

NMR spectra were recorded on a Bruker Ultrashield 500 spectrometer equipped with a 5-mm TCI cryoprobe (^1^H at 500 MHz, ^13^C at 125 MHz). For HMBC, HSQC, and COSY experiments, standard pulse programs were used and HMBC experiments were optimized for ^2,3^J_C-H_ = 6 Hz. All observed chemical shift values (δ) are given in ppm and coupling constant values (*J*) in Hz. Chemical shifts of the remaining nondeuterated methanol solvent signals at δ_H_ 3.31 ppm and δ_C_ 49.2 ppm were used as reference signals for spectrum calibration. To increase sensitivity, the measurements were performed in a 5-mm Shigemi tube (Shigemi Inc., Allison Park, PA, USA). Processing and data evaluation were carried out using the ACD/NMR Workbook Suite Enterprise 2019.

### Marfey’s method to elucidate the stereochemistry of the arginine moiety.

A total of 100 μg of sesbanimide R was dissolved in 100 μl of 6 N HCl and incubated for 45 min at 110°C. It was subsequently dried under N_2_ stream and redissolved in 110 μl of dH_2_O. This was then split into 2 50-μl portions, and 20 μl of l-FDLA or d-FDLA and 20 μl of NaHCO_3_ were added. The reaction mixture was shaken at 700 rpm and 40°C for 2 h, and then the reaction was stopped with the addition of 10 μl of 2 N HCl. The reaction mixture was then diluted with 300 μl of acetonitrile, centrifuged, and analyzed using LC-MS system 1b. The same reaction and measurement, without the hydrolysis, were performed with l- and d-arginine as a reference. The retention times of the derivatized standards were compared to those of the derivatized samples to assign the stereochemistry.

### Saponification of sesbanimide R.

A total of 50 μg of sesbanimide R was dried and redissolved in 100 μl of 2 M NaOH. The reaction was stopped instantly by adding 200 μl of 1 M HCl, and an aliquot of the solution was diluted 1:5 with acetonitrile and analyzed on LC-MS system 1a.

### Cytotoxicity assays with HCT-116, HepG2, KB3.1, and A549 cells.

The cell lines were obtained from the German Collection of Microorganisms and Cell Cultures (Deutsche Sammlung für Mikroorganismen und Zellkulturen [DSMZ]) and cultured under conditions recommended by the depositor. Cells were grown and diluted to 5 × 10^4^ per well of 96-well plates in 180 μl of complete medium. After 2 h of equilibration, the cells were treated with a serial dilution of sesbanimide R in methanol. A total of 20 μl of 5 mg/ml of thiazolyl blue tetrazolium bromide (MTT) in phosphate-buffered saline (PBS) was added to each well after growing the cells for 5 days. The cells were further incubated for 2 h at 37°C before the supernatant was discarded. Subsequently, the cells were washed with 100 μl of PBS and treated with 100 μl of 2-propanol/10 N HCl (250:1) to dissolve formazan granules. Cell viability was measured as a percentage relative to the respective methanol control by measuring the absorbance at 570 nm with a microplate reader (Tecan Infinite 200 PRO). GraphPad Prism was used for sigmoidal curve fitting to determine the IC_50_ values as well as the calculation confidence intervals.
