# Supplementary material for: Sesbanimide R, a Novel Cytotoxic Polyketide Produced by Magnetotactic Bacteria
Source: mBio. 2021 May 18;12(3):e00591-21. doi: 10.1128/mBio.00591-21 (PMC8262917; doi:10.1128/mBio.00591-21)
Supplement: TABLE S1 [file mbio.00591-21-st001.docx]

Table S1: Putative secondary metabolites gene clusters present in the genome of *Magnetospirillum gryphiswaldense* with locus tag and size.

| **Gene Clusters** | **Locus tags** |
| --- | --- |
| Lasso peptide | MSR-1_06390-06890 |
| Aryl polyene | MSR-1_09890-10640 |
| Homoserine lactone | MSR-1_16040-16260 |
| Trans-AT PKS | MSR-1_15520-15810 |
