# Supplementary figures and images for: Sesbanimide R, a Novel Cytotoxic Polyketide Produced by Magnetotactic Bacteria
Source: mBio. 2021 May 18;12(3):e00591-21. doi: 10.1128/mBio.00591-21 (PMC8262917; doi:10.1128/mBio.00591-21)

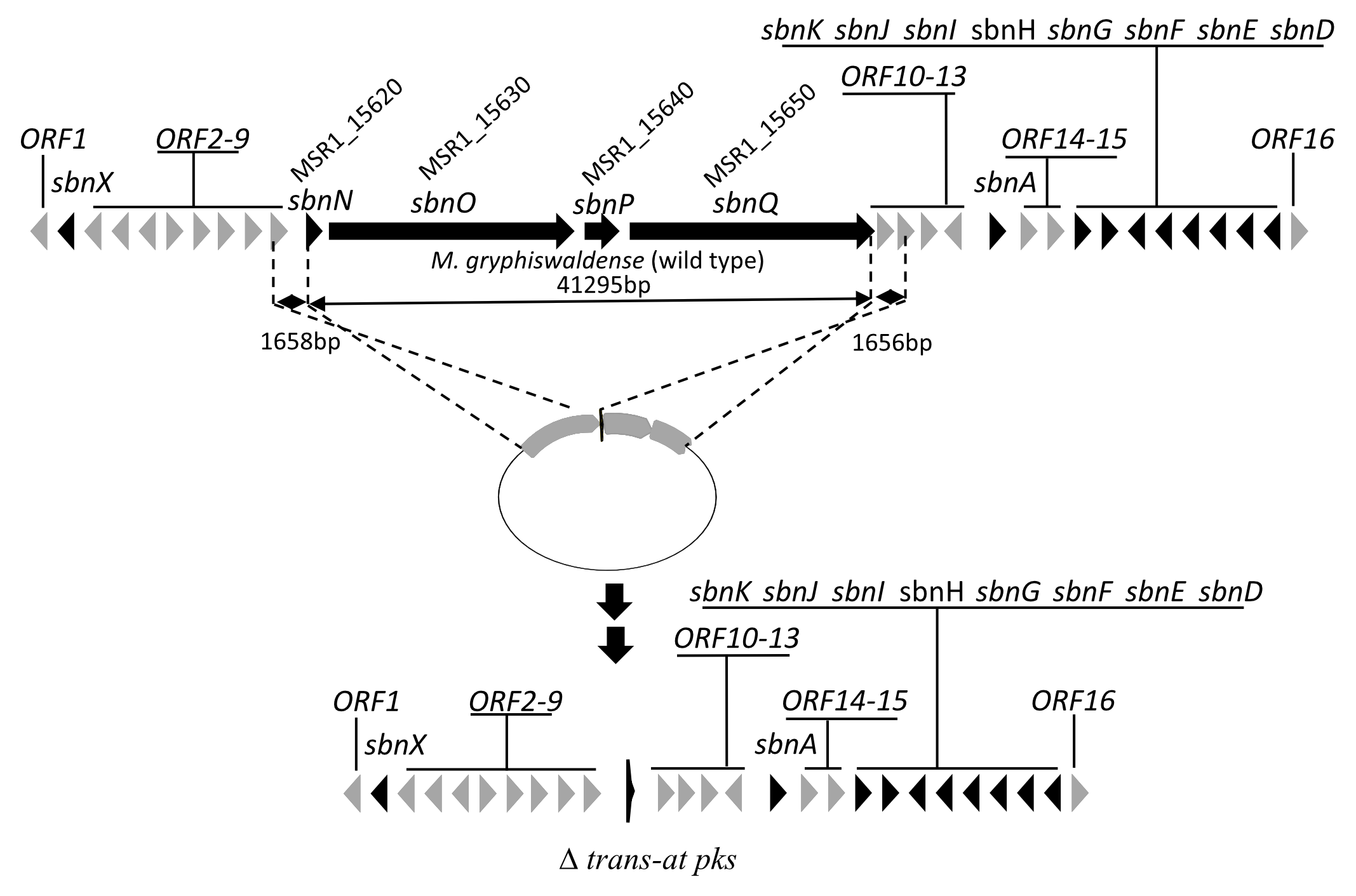

Supplement: FIG S1 [file mbio.00591-21-sf001.tif]

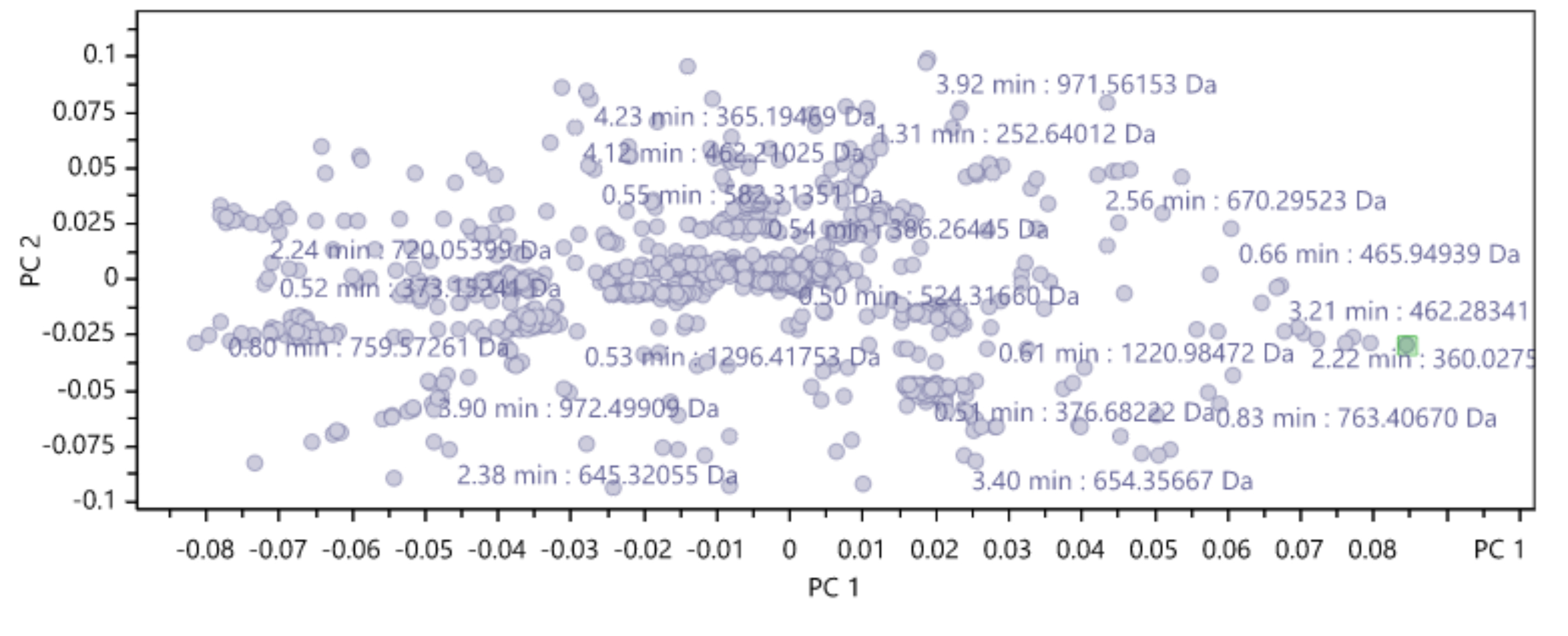

Supplement: FIG S2 [file mbio.00591-21-sf002.tif]

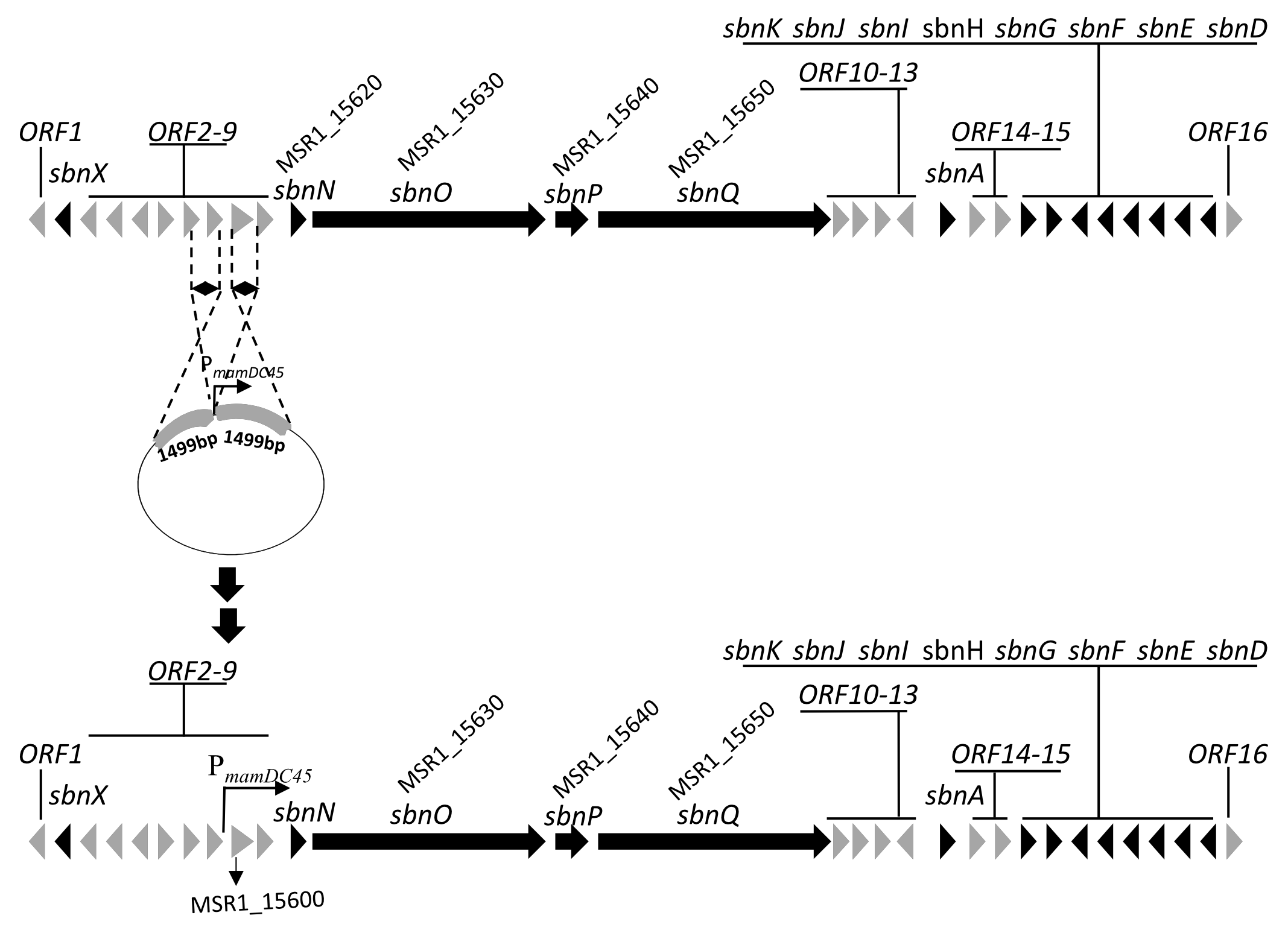

Supplement: FIG S3 [file mbio.00591-21-sf003.tif]

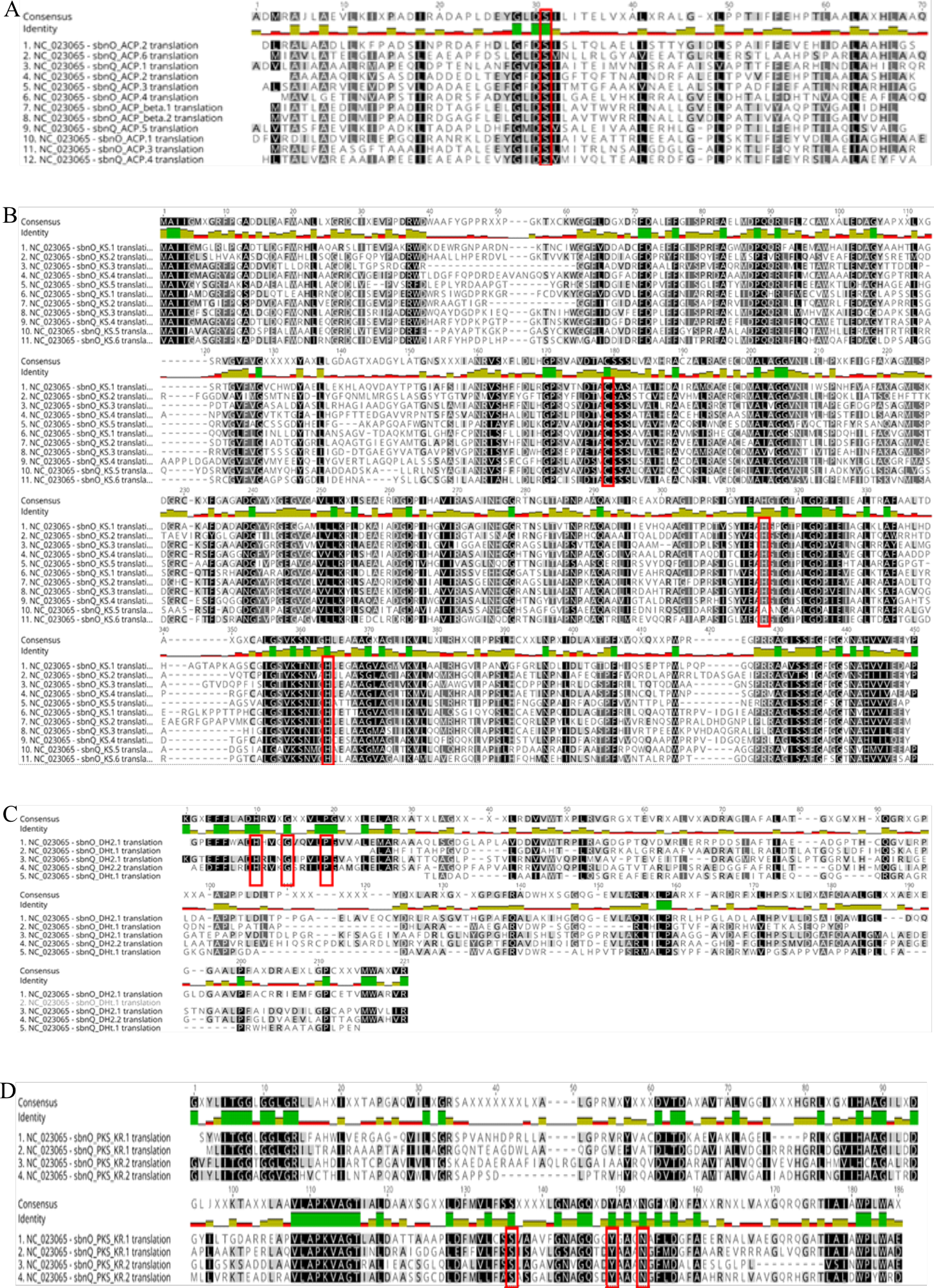

Supplement: FIG S4 [file mbio.00591-21-sf004.tif]

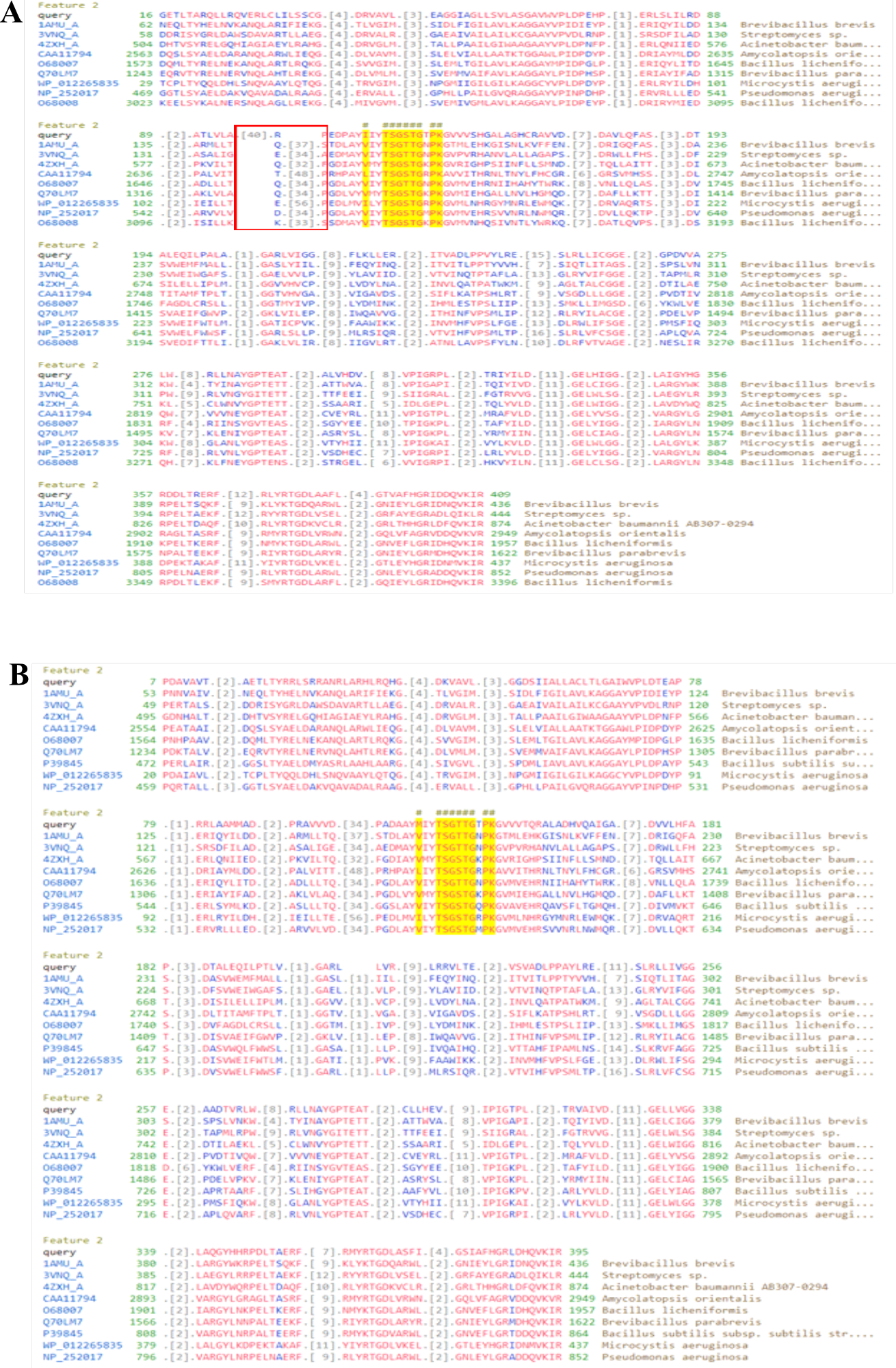

Supplement: FIG S5 [file mbio.00591-21-sf005.tif]
