# Supplementary material for: Sesbanimide R, a Novel Cytotoxic Polyketide Produced by Magnetotactic Bacteria
Source: mBio. 2021 May 18;12(3):e00591-21. doi: 10.1128/mBio.00591-21 (PMC8262917; doi:10.1128/mBio.00591-21)
Supplement: TABLE S2 [file mbio.00591-21-st002.docx]

**In silico analysis of the sesbanimide biosynthetic gene cluster from *Magnetospirillum gryphiswaldense***

Table S2: Annotation of the sesbanimide gene cluster in Magnetospirillum gryphiswaldense and comparison the sesbanimide cluster in PHM037 and PHM038

|  | **Putative function/ homologue** | **Accession number of closest protein homologue** | **Cover/Pairwise identity [%]** | **Corresponding gene in PHM037/ PHM038** |
| --- | --- | --- | --- | --- |
| Orf1 | CoA transferase | WP_024079852 | 100/100 | - |
| Orf2 | response regulator | WP_024079854 | 100/100 | - |
| Orf3 | response regulator receiver modulated diguanylate cyclase | CDK98834 | 100/100 | - |
| Orf4 | Hpt domain-containing protein | WP_024079856 | 73.5/100 | - |
| Orf5 | diguanylate cyclase | WP_024079857 | 90.4/100 | - |
| Orf6 | amino acid ABC transporter substrate-binding protein | WP_024079858 | 100/100 | - |
| Orf7 | response regulator | WP_024079859 | 100/100 | - |
| Orf8 | cyclic peptide export ABC transporter | WP_024079860 | 100/100 | SbnL |
| Orf9 | cyclic peptide export ABC transporter | WP_024079861 | 100/100 | SbnM |
| Orf10 | Band 7 protein | CDK98845 | 100/100 | SbnR |
| Orf11 | ABC transporter substrate-binding protein | WP_024079867 | 100/100 | SbnS |
| Orf12 | hypothetical protein | WP_024079868 | 100/100 | SbnT |
| Orf13 | DUF-697 domain-containing protein | WP_024079869 | 94.7/100 | SbnU |
| Orf14 | putative lysine-arginine-ornithine-binding periplasmic protein | CDK98850 | 100/100 | - |
| Orf15 | cation:dicarboxylase symporter family transporter | WP_024079872 | 88.7/99.8 | - |
| Orf16 | metallophosphoesterase | WP_041633527 | 100/100 | SbnC |
| SbnA | acyltransferase domain-containing protein | WP_158497748 | 73.8/99.7 | SbnA |
| SbnD | Fkbm family methyltransferase | WP_158497749 | 87.2/100 | SbnD |
| SbnE | cytochrome P450 | WP_024079879 | 100/100 | SbnE |
| SbnF | hydroxymethylglutaryl-CoA synthase family protein | WP_024079878 | 100/100 | SbnF |
| SbnG | acyl carrier protein | WP_024079877 | 100/100 | SbnG |
| SbnH | decarboxylase | WP_024079876 | 100/100 | SbnH |
| SbnI | enoyl-CoA hydratase/ isomerase | WP_024079875 | 100/100 | SbnI |
| SbnJ | asparagine synthase (glutamine-hydrolyzing) | WP_024079874 | 100/100 | SbnJ |
| SbnK | acyl carrier protein | WP_106002060 | 100/96.3 | SbnK |
| SbnN | ACP  S-malonyltransferase | WP_024079862 | 100/100 | SbnN |
| SbnO | SDR family NAD(P)-dependent oxidoreductase | WP_024079863 | 100/100 | SbnO |
| SbnP | monooxygenase | OJX77727 | 99.5/87.0 | SbnP |
| SbnQ | non-ribosomal peptide synthetase | WP_024079865 | 100/100 | SbnQ |
| SbnX | acyl-CoA dehydrogenase | WP_024079853 | 100/100 | SbnX |
