# Supplementary material for: Sesbanimide R, a Novel Cytotoxic Polyketide Produced by Magnetotactic Bacteria
Source: mBio. 2021 May 18;12(3):e00591-21. doi: 10.1128/mBio.00591-21 (PMC8262917; doi:10.1128/mBio.00591-21)
Supplement: TABLE S3 [file mbio.00591-21-st003.docx]

Table S3: Substrate specificities as predicted by the TranATor tool and sorted according to E-value. The predictions that fit the structure and biosynthesis proposal are highlighted in bold.

|  | **prediction** | **e value** | **score** |
| --- | --- | --- | --- |
| KS1 (sbnO) | Clade 95 various specificities  Clade 109 completely reduced  **Clade 8 unusual starter (AMT/Succinate)**  Clade 136 β D-OH  Clade 96 various specificies (mainly α-Me) | 1.1E-179  1.0E-176  2.8E-217  2.8E-178  2.0E-177 | 590.7  580.7  714.5  585.8  583.2 |
| KS2 (sbnO) | Clade_64 non-elongating (double bonds (mostly z-configured))  Clade 5 amino acids (oxa/thia)  **Clade 82 double bonds (mostly e-configured)**  **Clade 115 β-keto or double bonds**  **Clade 99 double bonds (e-configured)** | 9.1E-218  2.9E-151  5.8E-151  7.8E-149  4.4E-147 | 715.9  497.0  495.9  488.8  483.4 |
| KS3 (sbnO) | Clade 95 various specificities  Clade 109 completely reduced  Clade 96 various specificies (mainly α-Me)  **Clade 12 vinylogous chain branching**  Clade 136 β D-OH | 2.1E-149  1.4E-141  4.3E-166  2.5E-155  5.0E-153 | 490.9  464.9  545.8  510.2  502.6 |
| KS4 (sbnO) | **Clade_7 ß D-OH**  **Clade_110 ß D-OH or double bonds (e-configured)**  **Clade_140 β D-OH**  **Clade_137 β D-OH**  **Clade_62 β D-OH (some with α L-Me)** | 1.2E-207  8.7E-212  9.2E-209  8.9E-207  1.6E-201 | 683.1  696.5  686.7  680.0  662.6 |
| KS5 (sbnO) | Clade_68 α L-OH/Me β D-OH  Clade_104 ß OMe or ß Me double bond  **Clade_21 α Me reduced/keto/D-OH**  **Clade_74 α Me reduced/keto/D-OH**  **Clade_23 α-Me** | 8.6E-174  8.4E-170  2.5E-194  7.3E-193  5.9E-180 | 571.1  557.7  638.7  633.9  591.3 |
| KS6 (sbnO) | Clade_104 ß OMe or ß Me double bond  Clade_86 α-L-Me red or OH  Clade_14 exomethyl/exoester  Clade_2 α-Me shifted double bond or OH  **Clade_73 exomethylene** | 1.9E-177  6.7E-169  9.3E-196  3.6E-174  1.1E-169 | 582.9  554.9  643.5  572.2  557.3 |
| KS7 (sbnQ) | **Clade_35 oxidative rearrangement**  Clade_95 various specificities  Clade_25 completely reduced  Clade_96 various specificies (mainly α-Me)  Clade_136 β D-OH | 4.7E-214  9.4E-167  8.3E-173  3.7E-172  8.9E-169 | 704.0  548.0  567.8  565.8  554.5 |
| KS8 (sbnQ) | Clade_95 various specificities  **Clade_25 completely reduced**  Clade_108 shifted double bonds  Clade_96 various specificies (mainly α-Me)  Clade_136 β D-OH | 2.6E-181  1.2E-201  5.2E-191  9.3E-188  2.2E-173 | 596.0  662.9  627.9  617.2  569.7 |
| KS9 (sbnQ) | **Clade_25 completely reduced**  Clade_108 shifted double bonds  Clade_96 various specificies (mainly α-Me)  Clade_11 shifted double bonds  Clade_136 β D-OH | 9.4E-217  2.9E-204  1.2E-190  6.5E-189  5.4E-185 | 712.6  671.6  626.7  621.0  607.9 |
| KS10 (sbnQ) | **Clade_82 double bonds (mostly e-configured)**  **Clade_125 double bonds (e-configured)**  **Clade_129 double bonds (e-configured)**  **Clade_101 double bonds**  **Clade_99 double bonds (e-configured)** | 1.5E-227  6.4E-223  6.5E-213  9.2E-213  9.9E-213 | 748.3  733.0  700.1  699.5  699.7 |
| KS11 (sbnQ) | **Clade_76 non-elongating (double bonds)**  **Clade_142 non-elongating (various)**  Clade_101 double bonds  Clade_90 β-keto or double bonds  Clade_115 β-keto or double bonds | 1.7E-179  2.7E-167  1.6E-162  4.1E-161  8.7E-160 | 589.9  549.6  534.0  529.5  524.9 |
