# Supplementary material for: Sesbanimide R, a Novel Cytotoxic Polyketide Produced by Magnetotactic Bacteria
Source: mBio. 2021 May 18;12(3):e00591-21. doi: 10.1128/mBio.00591-21 (PMC8262917; doi:10.1128/mBio.00591-21)
Supplement: TABLE S4 [file mbio.00591-21-st004.docx]

Table S4: Putative open reading frames (ORFs) encoding polyketide synthases (PKSs), non-ribosomal peptide synthetases (NRPSs), or hybrid in PKS and NRPS gene clusters from different magnetotactic bacteria. *indicates that genome assembly of these species is not complete, and the number of Open Reading Frames (ORFs) might be variable in the final analysis.

| **Species** | **strain** | **Class** | **PKS** | **NRPS** | **Hybrid** | **Trans-AT PKS** |
| --- | --- | --- | --- | --- | --- | --- |
| ***Magnetospirillum gryphiswaldense*** | MSR-1 | α | 0 | 0 | 0 | 1 Trans-AT PKS |
| ***Magnetospirillum magneticum*** | AMB-1 | α | 1 T1PKS | 1 NRPS-like | 0 | 0 |
| ***Magnetospira* sp.** | QH-2 | α | 0 | 1 NRPS | 0 | 0 |
| ***Magnetospira* sp.** | ME-1 | α | 1 T1PKS | 1 NRPS-like | 0 | 0 |
| ***Magnetovibrio blakemorei **** | MV-1 | α | 0 | 0 | 0 | 1 Trans-AT PKS |
| ***Magnetospirillum* sp.** | XM-1 | α | 1 T1PKS | 1 NRPS-like | 0 | 0 |
| ***Magnetofaba australis **** | IT-1 | α | 1 T1PKS | 0 | 0 | 0 |
| ***Magnetospirillum magnetotacticum **** | MS-1 | α | 1 T1PKS | 1 NRPS-like | 0 | 0 |
| ***Magnetospirillum* sp. *** | SO-1 | α | 1 T1PKS | 1 NRPS-like | 0 | 0 |
| ***Magnetospirillum marisnigri **** | SP-1 | α | 1 T3PKS | 1 NRPS-like | 0 | 0 |
| ***Magnetospirillum* sp. *** | mag 15-1 | α | 1 T1PKS | 1 NRPS-like | 0 | 1 Trans-AT PKS |
| ***Magnetospirillum* sp. *** | 64-120 | α | 0 | 0 |  | 1 Trans-AT PKS |
| **Magneto-ovoid bacterium** | MO-1 | α | 0 | 1 NRPS / 1 NRPS-like | 0 | 0 |
|  |  |  |  |  |  |  |
| ***Desulfovibrio magneticus*** | RS-1 | δ | 0 | 1 NRPS-like | 0 | 0 |
| ***Desulfamplus magnetovallimortis **** | BW-1 | δ | 2 T1PKS | 0 | 0 | 0 |
| ***Candidatus* Magnetoglobus multicellularis str.*** | Araruama | δ | 1 T1PKS | 5 NRPS / 2 NRPS-like |  | 2 Trans-AT PKS like |
|  |  |  |  |  |  |  |
| **Ectothiorhodospiraceae bacterium** | BW-2 | γ | T2PKS | 1 NRPS-like | 2 NRPS/T1PKS |  |
| **Gamma Proteobacterium** | SS-5 | γ | 0 | 1 NRPS | 2 NRPS/T1PKS | 1 Trans-AT PKS |
|  |  |  |  |  |  |  |
| ***Candidatus* Magnetobacterium casensis *** | MYR-1 | Nitrospirae | 0 | 1 NRPS-like | 0 | 0 |
| ***Candidatus* Magnetobacterium bavaricum *** | TM-1 | Nitrospirae | 0 | 1 NRPS-like | 0 | 0 |
| ***Candidatus* Magnetoovum chiemensis *** | CS-04 | Nitrospirae | 1 T1PKS | 1 NRPS | 0 | 0 |
