# Supplementary material for: Sesbanimide R, a Novel Cytotoxic Polyketide Produced by Magnetotactic Bacteria
Source: mBio. 2021 May 18;12(3):e00591-21. doi: 10.1128/mBio.00591-21 (PMC8262917; doi:10.1128/mBio.00591-21)
Supplement: TABLE S5 [file mbio.00591-21-st005.docx]

Table S5: Strains, vectors and primers used in this study.

| **Strain or vector** | | | **Relevant characteristic (s)** | **Reference and/or source** |
| --- | --- | --- | --- | --- |
| **Strains** | | |  |  |
|  | *E. coli* | |  |  |
|  |  | DH5α | Host for cloning; F^+^ *endA1* *glnV44* *thi-1* *recA1* *relA1* *gyrA96* *deoR* *nupG* *purB20* φ80d*lacZ*ΔM15 Δ(*lacZYA-argF*)U169, hsdR17(*r_K_*^–^*m_K_*^+^), λ^–^ | (1) |
|  |  | WM3064 | Conjugation strain; *thrB1004* *pro* *thi rpsL* *hsdS* *lacZ*ΔM15 *RP4-1360* Δ(*araBAD*)*567* Δ*dapA1341*::[*erm* *pir*] | William Metcalf at UIUC |
|  | *M. gryphiswaldense* | |  |  |
|  |  | Wild type | MSR-1 R3/S1; Rif^R^, Sm^R^ | (2) |
|  |  | Δ*trans-at-pks* | Core-biosynthetic genes of trans-AT PKS deletion strain  (MSR-1_15620-15650) | This study |
|  |  | P*_mamDC45_-trans-at-pks* | Strain with chromosomally inserted P*_mamDC_*_45_ promoter in front of MSR-1_15600 | This study |
| **Vectors** | | |  |  |
|  | pORFM | | universal in-frame deletion/in-frame fusion vector for GalK-based counterselection; *npt* *galK* *tetR mob*RK2 | (3) |
|  | pORFM-Δ*trans-at-pks* | | Vector for chromosomal deletion of core-biosynthetic genes of trans-AT PKS gene cluster (MSR-1_15620-15650) | This study |
|  | pORFM-*P_mamDC45_*_-_*trans-at-pks* | | Vector for insertion of a promoter 1xPmamDC45-oRBS in front of MSR-1_15600 | This study |

**Primers**

| **No. (RPA)** | **Primer name** | | **Sequence 5’-3’** |
| --- | --- | --- | --- |
| ***Site-specific chromosomal deletion/insertion by homologous recombination*** | | | |
| 595 | F1_del_Trnpks-nrps | gtcattactggatctatcaacaggagtcctgcagtaggatgagcatcgccgctttcctggg | |
| 596 | R1_del_Trnpks-nrps | gctggatcggttagcccgaggctttcatgcatggcctccttcgc | |
| 597 | F2_del_Trnpks-nrps | ggaggccatgcatgaaagcctcgggctaaccgatccagcataatatg | |
| 598 | R2_del_Trnpks-nrps | gcggcagcgtgaagctagcatcactagtctagcgcagcaggtcatcgatggagcgg | |
| 599 | sq_Rev1_Trnpks_nrps | cccatacaggcggtcaaacag | |
| 600 | sq_For2_Trnpks_nrps | cgaggtggtgttcgtggtc | |
| 601 | sq_Rev2_Trnpks_nrps | ggcttttggcgatgatctgc | |
| 602 | sq_For3_Trnpks_nrps | accgctacatcatcgtcgac | |
| 937 | F1_Pro_in_PKS_NRPS | caggaaagacttaagctgcagtagggcccgggtgatggtcgcc | |
| 938 | R1_Pro_in_PKS_NRPS | gagaactaagagctagtaaagcgaaaaagtcttacttgtcttgtccggcg | |
| 939 | F2_Pro_in_PKS_NRPS | cgccatcgccggacaagacaagtaagactttttcgctttactagctc | |
| 940 | R2_Pro_in_PKS_NRPS | ggtgctgacgagacgaagaacatgcatatgctgatctcctaagcttcgc | |
| 941 | F3_Pro_in_PKS_NRPS | ccctgcgaagcttaggagatcagcatatgcatgttcttcgtctcgtcagc | |
| 942 | R3_Pro_in_PKS_NRPS | ctctagactaaagcttatcgaattcctagccagaaccgtatagaacaattcg | |
| 943 | Ck_barA_CT_R | catgtcgttgccgtcaagc | |
| 944 | Ck_P_PKS_NRPS_F | gaccttgtacgaatgctgcc | |
| 945 | Ck_P_PKS_NRPS_R | cgcatcaattcgtggtccag | |
| 946 | Ck_yojil1_NT_F | gcaccttggaaaatctcggc | |
| 484 | sq_bk_pORFM_F | gccactcatcgcagtctagc | |
| 485 | sq_pORFM_bk_Rev | tctgcggactggctttctac | |

References

1. Hanahan D. 1983. Studies on transformation of Escherichia coli with plasmids. Journal of Molecular Biology 166:557–580. doi:10.1016/s0022-2836(83)80284-8.

2. Schultheiss D, Kube M, Schüler D. 2004. Inactivation of the flagellin gene flaA in Magnetospirillum gryphiswaldense results in nonmagnetotactic mutants lacking flagellar filaments. Appl Environ Microbiol 70:3624–3631. doi:10.1128/AEM.70.6.3624-3631.2004.

3. Raschdorf O, Plitzko JM, Schüler D, Müller FD. 2014. A tailored galK counterselection system for efficient markerless gene deletion and chromosomal tagging in Magnetospirillum gryphiswaldense. Appl Environ Microbiol 80:4323–4330. doi:10.1128/AEM.00588-14.
